# Impact of mental imagery on enhancing surgical skills learning in novice’s surgeons: a pilot study

**DOI:** 10.1186/s12909-021-02987-z

**Published:** 2021-10-28

**Authors:** Tarik Souiki, Mohammed Benzagmout, Badreeddine Alami, Karim Ibn Majdoub, Imane Toughrai, Khalid Mazaz, Saïd Boujraf

**Affiliations:** 1 Clinical Neurosciences Laboratory, Faculty of Medicine and Pharmacy of Fez, Sidi Mohammed Ben Abdellah University, BP. 1893; Km 2.200, Sidi Hrazem Road, 30000 Fez, Morocco; 2 Department of Visceral Surgery E3, University Hospital Hassan II, Fez, Morocco

**Keywords:** Mental imagery, Surgical education, Hand-sewn intestinal anastomosis

## Abstract

**Objective:**

Mental imagery (MI) has long been used in learning in both fields of sports and arts. However, it is restrictively applied in surgical training according to the medical literature. Few studies have evaluated its’ feasibility and usefulness. The aim of this study is to assess the impact of mental imagery on surgical skills learning among novice’s surgeons.

**Material and methods:**

In this pilot prospective randomized comparative study; we recruited 17 residents and interns of surgery education curriculum. They were all included in their first semester of the curricula. Two groups were randomly designed. Group (a) including “Mental Imagery” volunteers (*n* = 9) which benefited from a mental imagery rehearsal exercise prior to physical practice, while the control group (b) (*n* = 8) didn’t underwent any MI process prior to surgery practice. Each participant of both groups was invited to perform an intestinal hand-sewn anastomosis on bovine intestine. Each procedure was evaluated and analyzed according to 14 qualitative criteria while each criterion was scored 0, 1 or 2 respectively assigned to the gesture was not acquired, gesture was performed with effort, or mastered gesture. The final score is 28 for those who master all 14 gestures. A non-parametric statistical comparison between the both studied groups was performed.

**Results:**

Both groups of surgery students demonstrated equivalent age, sex ratio, laterality, and surgical experience. The mean overall score is significantly higher in the MI group (a) (17.78; SD = 2.42) compared to the control group (b) (10.63, SD = 2.85). However, advanced analysis of individual assessment items showed significant statistical difference between both groups only in 6 out of 14 assessed items.

**Conclusion:**

Indeed, mental imagery will not be able to substitute the traditional learning of surgery for novice surgeons; it is an important approach for improving the technical skills acquisition and shortening the physical learning.

## Introduction

Mental imagery (MI) is defined as “the cognitive rehearsal of a given task in the absence of manifest physical movement” [1]. It is a technique allowing to mentally present a given gesture or action without any real execution [2]. This human brain capability is applied in gestural education. Thus, this category of mental exercise has been shown to improve performance in the acquisition of technical skills. Hence, it is well-recognized and validated as educational tool in several fields such as sports, music and aviation [3–5].

Currently, difficulties are experienced in traditional surgical teaching and training. Hence, MI has been suggested by several authors as a complementary method to enhance surgical training quality and improve the education time [6]. Despite promising potential of MI, the validity of the method remains understudied and not sufficiently reported in medical literature.

In this study, we conducted a randomized trial involving first-semester residents and interns of general surgery; we targeted to assess the impact of a mental imagery exercises on the surgical gestures learning consisting of performing a hand-sewn intestinal anastomosis (HIA), that is a common surgical procedure used by general surgeons. It consists of establishing connection between two intestinal segments. The technical defects of this basic surgical procedure in digestive surgery exposes patient to potential septic risks whish might cause a dramatic complications including anastomosis leaks, fistula, and peritonitis. We were particularly interested in providing a mental imagery protocol immediately after learning and immediately before the evaluation stage.

## Material and methods

**Fig. 1:**
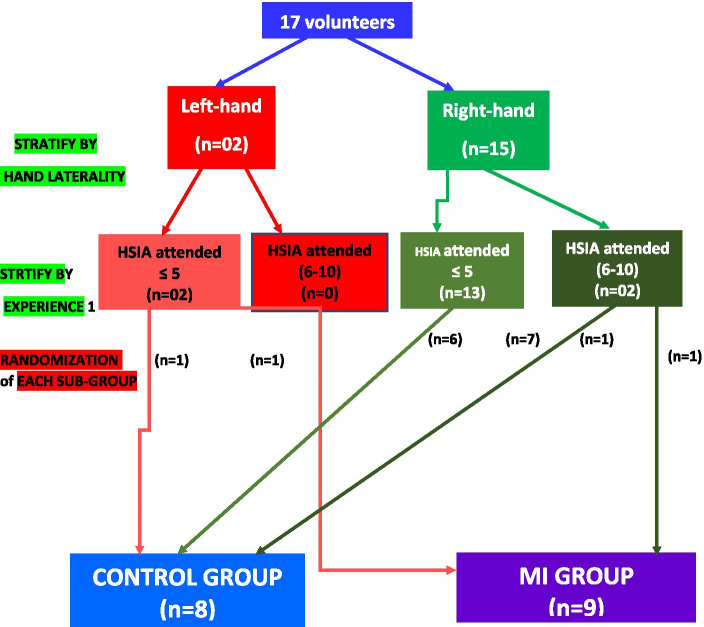
Stratification and randomization process applied in the study

**Fig. 2:**
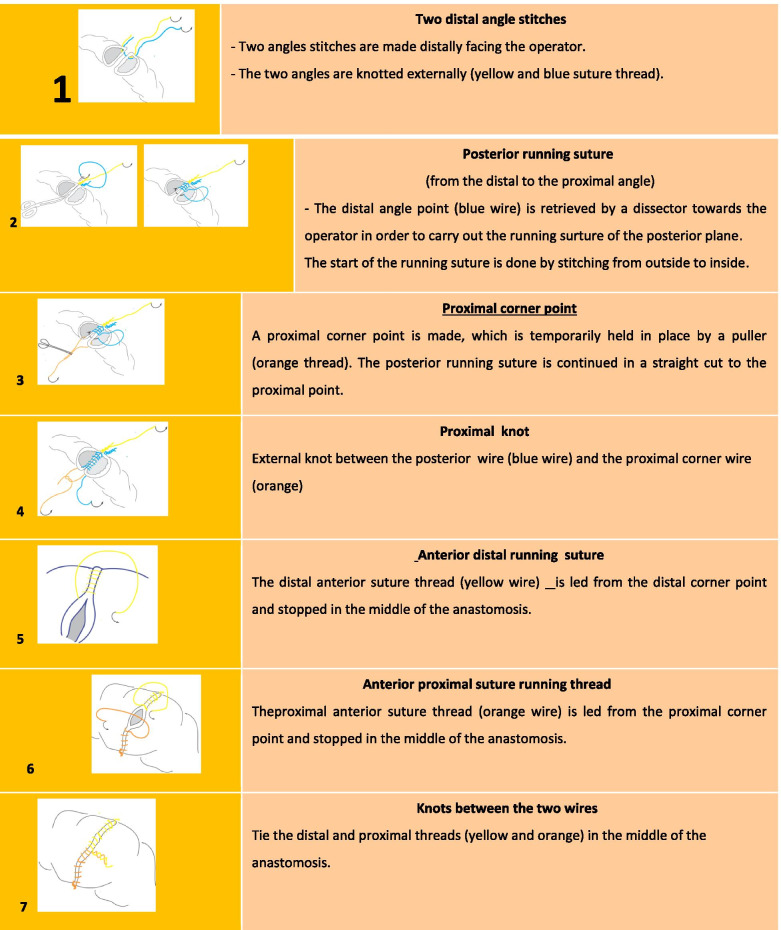
Hand-sewn intestinal anastomosis protocol

This prospective randomized comparative study targeted to assess the impact of MI in the learning of surgical skills by first semester surgical interns and residents. Twenty residents and interns enrolled in the first semester of general surgery were recruited to participate to a workshop to learn HIA. They are all in their sixth month of the first semester. Their participation was done on a voluntary basis. Thus, the objective of the participants was to perform HIA while full filling the well-known and standard surgical protocol of the technique. All participants were fully new to introduce the HIA technique. They have had already attended procedures involving HIA in the operating theatre, but never performed the surgical gesture themselves. None of the volunteers have been verified to use the MI technique before. All participants, thus, had the same level of practice. The initial plan included 20 residents; however, 3 participants were unable to be available due to their hospital duties in the stage of HIA workshop. Therefore, the final number of participants has decreased to 17. Initially, all subjects completed a baseline questionnaire to determine demographic characteristics including age, sex, numbers of HSIA attended (≤ 5 or 6 – 10) and hand laterality (right or left). The recruited populations were randomly split in two groups: group (a) that received MI training and control group (b). The randomization was carried out using the sealed opaque envelopes technique. Given the small sample size and imbalanced allocution to each group regarding certain determining parameters, such as the number of HSIA attended and hand laterality. Hence, we have stratified our sample while considering these last two variables. Finally, among 17 randomized participants, subsamples consisted of 9 participants assigned to MI arm and 8 participants that were assigned to the control arm. Participant’s enrollment and randomization implementation were allocated in sequence that was achieved by an investigator who is not involved in the post-procedure evaluation. Figure 1 is illustrating the randomization and the stratification processes achieved. In a first stage of the study protocol, all participants followed a 20-min presentation outlining the various key elements of performing a manual digestive anastomosis according to an established protocol of 7 steps (Fig. 2). A video demonstration of 5 min was designed to well elucidate the surgical gesture steeps; this video was shown and commented upon after the end of the presentation. A paper document including the protocol of the HIA procedure was provided to all participants. In a second step of the HIA learning protocol, the students were divided in two groups.

- The “control” group (b) was invited to move to another room to freely review the technique in their paper documents within 45 min allocated time.
- The “MI” group (a) benefited initially from a 5-min relaxation session including relax seating, body relaxing, deep breathing in a calm environment. This was followed by introducing the MI technique during 10 min where the teacher “mentalizes” with the participants each of the 7 steps of the HIA surgical procedure. Then, each participant of the MI group (a) was asked to perform mental imagery rehearsal of the entire surgical procedure within 30 min. The consultation of the paper document available was authorized whenever necessary to find the sequences required to acquire the technique. The whole procedure therefore lasted 45 min which is identical to the time allocated to the control group (b).

Then, all participants of both groups were gathered in the same laboratory room. Each student was asked to perform a manual digestive anastomosis using two segments of bovine colon. The performance of each student was evaluated by the same examiner, this examiner did not know any information about the participant’s background such as belonging to any of “control” or “mental imagery” groups. The evaluation focused on 14 qualitative criteria which are the key elements required to acquire the HIA gesture. The studied criteria were particularly emphasized in the paper document and on the video. Each criterion was rated 0, 1 or 2 respectively depending on whether the gesture was not acquired, whether it was performed with effort, or mastered. Besides, a score of 28 was attributed to the participants who mastered all the 14 HIA surgical gestures reported in Table. 1.

**Table 1:**
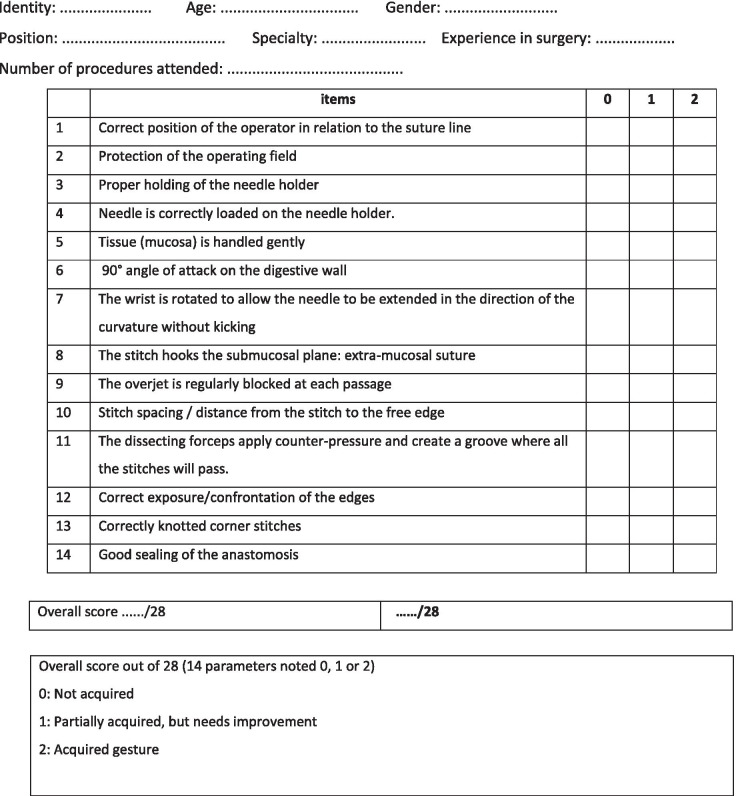
HIA assessment grid (max score: 28)

**Fig. 3:**
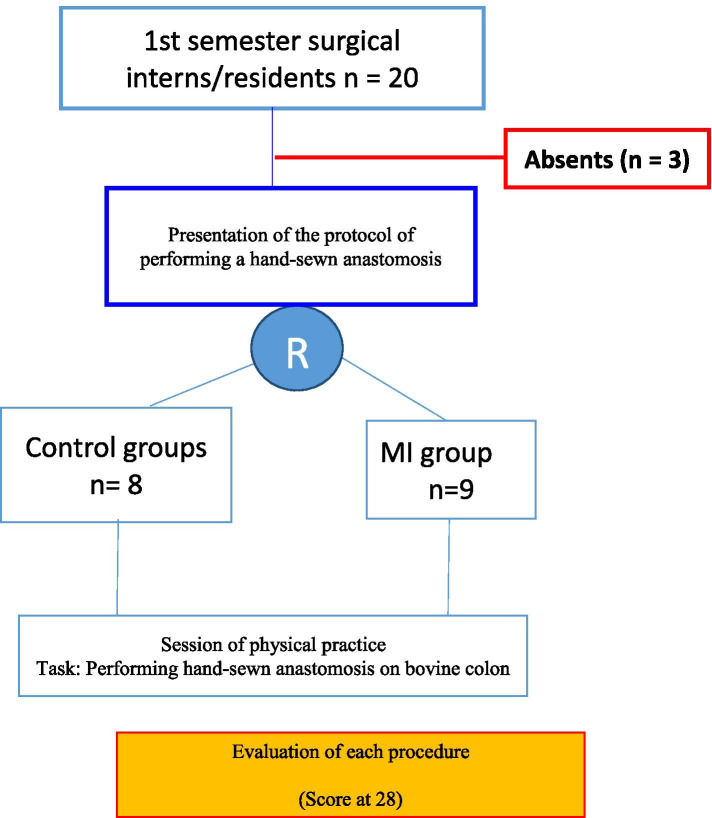
Study design used

Figure 3 summarizes the major key concept of the designed study. We used standard Microsoft Office Excel for data entry while statistical analysis was performed using SPSS 2.0. Besides, comparison of means and percentages was done using the Mann-Whitney nonparametric test. A significant difference was retained for a *p* < 0.05.

## Results

The average age of all participants was 29 years-old ranging from 26 to 32 years-old. The participants sample consisted of 14 men and 3 women consisting of 10 residents and 4 interns allocated in visceral surgery while 3 more residents were allocated in urological surgery. None of the participants have had performed HIA before. However, most of recruited participants have had attended HIA in the operating room. The difference in the number of previously observed gestures was not statistically significant between the two groups. Table 2 summarizes the different characteristics of the population studied in both groups.

**Table 2:** Participant’s characteristics

Characteristics	Control Group	MI Group	***P***
**Age**	27.78	28.13	0.7
**Sex:** Female/Male	1/7	2/7	0.547
**Surgical experience (in months)**	6	6	1
**Number HIA attended**			
**≤ 5**	7	8	1
**6–10**	1	1	
**Hand laterality (**Right/Left**)**	7/1	8/1	1

**Fig. 4:**
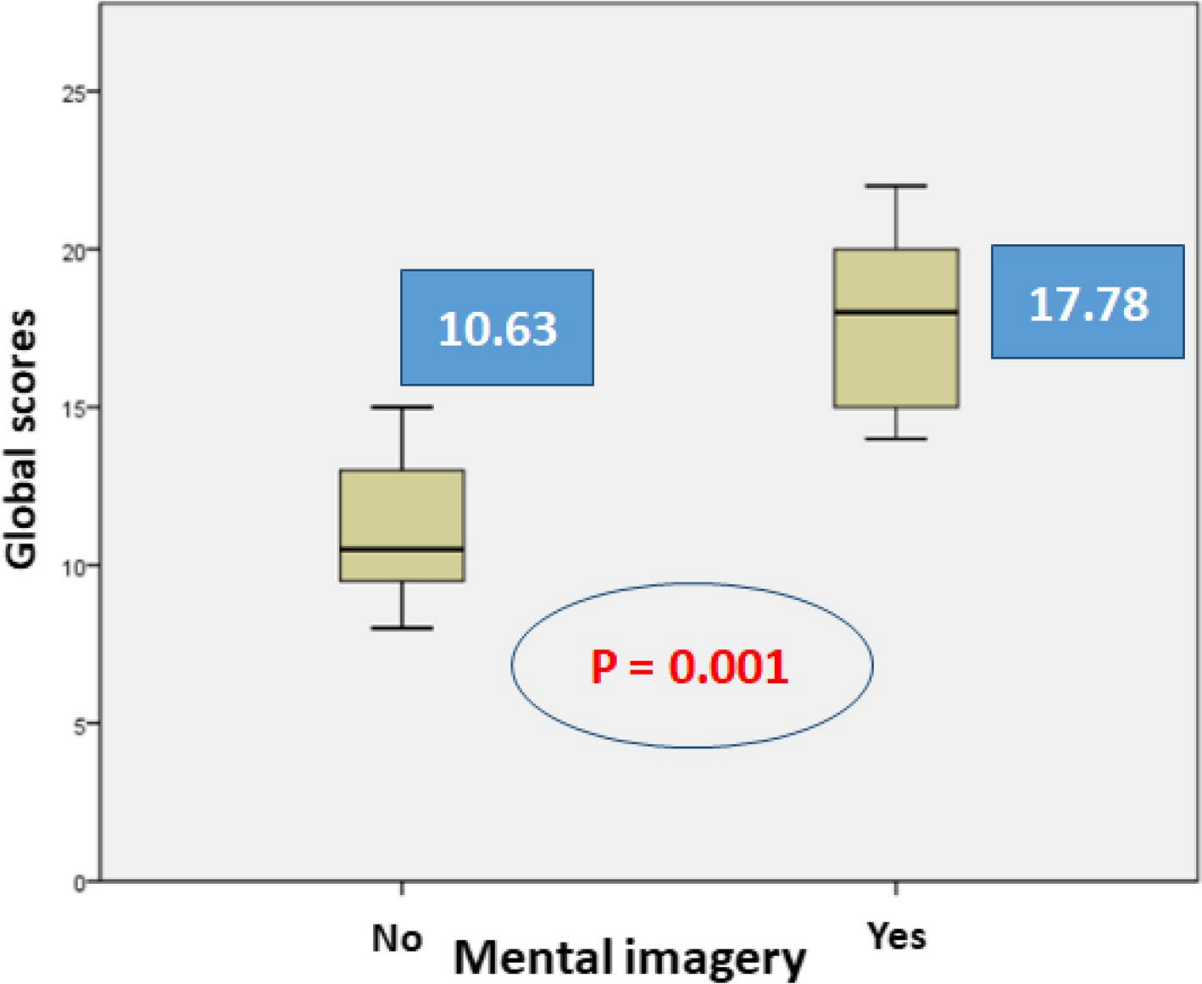
Global scores for MP group and control group

The group (a) that used MI technique recorded a mean score of 17.78 out of 28 (with *n* = 8, standard deviation = 2.42), while the control group (b) recorded a score of 10.63 (with *n* = 9, standard deviation = 2.85). These two means are statistically different (*p* = 0.001). Figure 4 illustrates extensively the reported result.

**Fig. 5:**
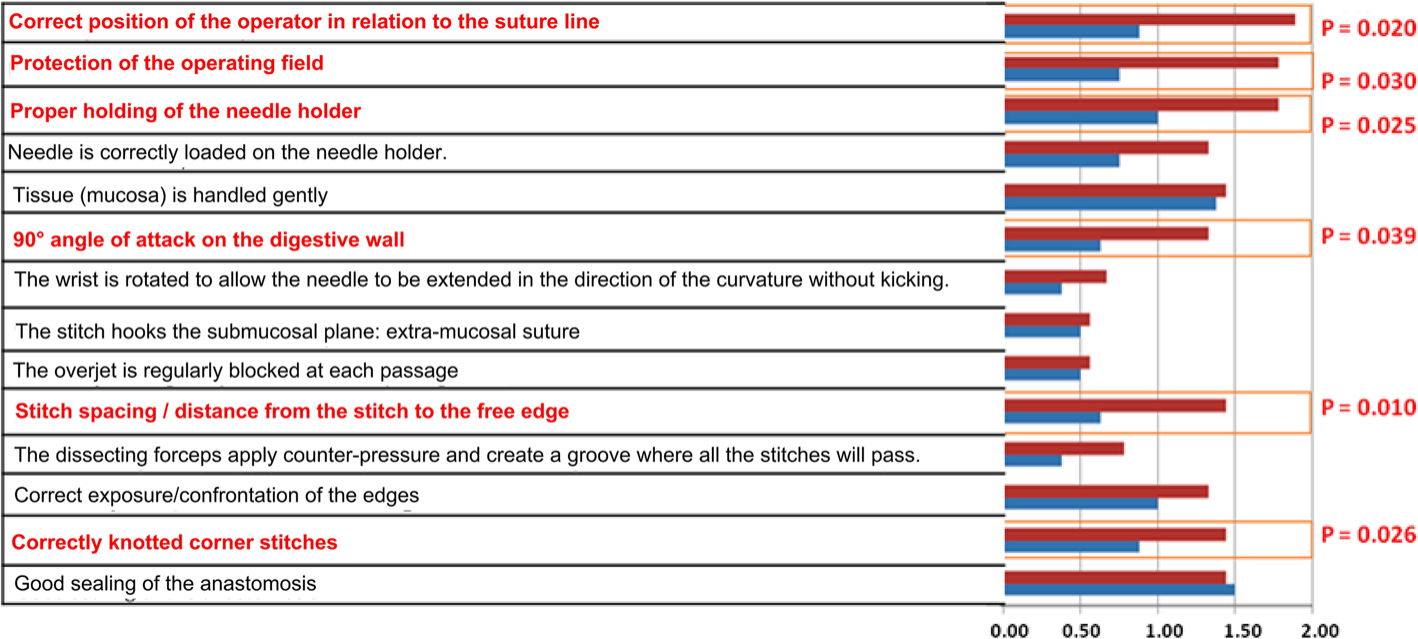
Comparison of the different items between the two groups

The advanced analysis consisted of comparing the mean score of each item of both groups. The results showed that scores of MI group (a) are always higher compared the control group (b). However, the significant differences are mainly limited to 6 items out of 14. Figure 5 illustrates the major results of this aspect.

The results of this empiric study reflected that MI exercised has provided an improvement in the acquisition of basic HIA gestures for novice’s surgeons. However, MI has not demonstrated any benefit of for all HIA studied gestures.

## Discussion

The concept of mental imagery was initiated a century ago and many theories have attempted to explain the mechanisms and effects on learning cognition. In 1931, Jacobson et al. observed the presence of fine peripheral muscle activity associated with the practice of mental imagery (MI) [7]. Thus, imagining the flexion of a right arm is demonstrated to be associated with a fine contraction of the muscle fibers of the same arm. This finding allowed to conclude that MI is associated with the activity of given motor neurons. But, the scientific rational for this so-called “neuromuscular” theory is currently shown controversial in recent studies. Guillot et al. demonstrated the occurrence of measurable electromyography activity simultaneously with the practice of MI [8]. This result was opposed by Gentilli et al. who did not objectify any modification of in the EMG recording during the mental practice [9]. Besides to the neuromuscular theory, more recent so-called cognitive theory stated that MI facilitates the central coding allowing to restructure and strengthen the cognitive representations [10, 11]. Currently, the results of functional MRI and positron emission tomography (PET) studies supported this hypothesis [12]. Both methodological studies showed that there are an activation of similar brain areas during an imagined movement compared to physically performed movement [13]. These studies stated a “neurofunctional equivalence” between imagined and physically achieved movement [14]. Authors reported an observation confirming the relation between effectively realized and imagined movements while both types of are achieved in similar time duration, they confirmed the Isochrony principle [15, 16].

Although the neuroscientific basis is not fully elucidated, MI has been shown to be effective in several areas. Indeed, it is widely used in high-level of professional sports [17]. According to studies conducted in this field, MI improves various aspects of sports performance including acceleration of technical skills acquisition, improvement of performance and motivation, better control of competition stress and faster recovery after injury. Besides, MI has also been applied in other fields such as music, dance and aviation [3–5, 18].

The well-established and used mode of surgical training and surgical teaching has long been based on apprenticeship model which is conducted using the “see one, do one, teach one” approach [6]. It is based on the immersion and gradual empowerment of the learner skills through repeated and supervised practice on patients in an operating room. However, this traditional method of teaching is currently facing economical, legal and ethical challenges. Unfortunately, there is an increasing evidences that the operating room is not an ideal learning environment for the first surgical skills training [6]. In fact, this educational environment is not learner-centered because considering the ethical reasons, the safety of the patient and the high caution that must be always taken over the surgeon’s learning. Furthermore, Expert surgeons may not be able to impart their expertise and skills to learners in a cost-effective way due to economic consideration such as a growing focus on operating room efficiency, time pressures and stressful conditions. All these increasingly growing constraints mean that hands-on supervised method in the operating room as an exclusive mode of learning is no sufficient and must be supplemented by other educational approaches allowing to extend learning outside the operating room. Thus, simulation centers have been arisen as alternative training field, but their widespread use remains limited due to the particular excessive cost and logistical requirements. Such limitations suggested to develop additional teaching techniques. In this constraining context, 20 years ago several authors suggested the use of MI as an adjunct teaching tool in surgical training. They were particularly inspired by MI’s success in sports [19]. The argument for this extrapolation is based on the existence of many obvious similarities of technical skill in the field of sport and surgery. In fact, technical gestures in these two areas are cognitively demanding; they require both concentration and precision, and they are often performed under very stressful conditions [20]. Moreover, it has been anecdotally reported that surgeons simulate the sequences of a surgical procedure prior to operation, particularly when the procedure is unusual or difficult. This constitutes an intuitive use of mental imagery without a conscious performance. It is the mind’s eye of surgeons. A study of 33 surgical operation performed by experienced surgeons with high risk of mortality showed that most of them attributed their intervention success to their mental readiness compared to technical readiness [21].

Several methodological limitations should be noticed before discussing the results of our study. First, the heterogeneity of participants’ level of training. In fact, although the participants have the same level of practice, they were interns at the end of their internship, residents in urology whose destination is not necessarily digestive surgery. Therefore, they do not have the same intrinsic motivation to learn this basic surgical technical of digestive surgery. In fact, the non-adaptability of the participants’ level in relation to the body movements evaluated is also one of the biases that was raised by Rao et al. in a review of recent literature [22]. Indeed, they noticed that negative studies without any demonstrated benefit of MI in this literature review are reporting surgical technical skills tested by medical students without any surgical experience. Second, the limitation is related to the assessment of the capacity of mental visualization. In fact, there are significant inter-individual differences in terms of imagery ability; individuals can easily build a mental representation of a given movement, while others find it more difficult [23]. Indeed, in the absence of an objective assessment of this ability, it cannot be concluded whether the results are related to a technical defect or to the participant’s mental capacity. It is therefore particularly interesting to test this ability to ensure the internal validity of any study evaluating the impact of MI. This bias is a real problem for most studies that have evaluated MI. To our knowledge, the study by Arora et al. [24] was the first to evaluated the capacity of mental imagery through a questionnaire (Fig. 5) that it adapted from “Movement Imagery Questionnaire (MIQ)” developed by hall et al. in the 1980s, and later abridged by Cumming et al. [25, 26]. Arora et al. demonstrated the validity and reliability in of the studied population before using it in a randomized controlled trial to assess the mental imaging capabilities for performing laparoscopic cholecystectomy [24]. Therefore, this MIQ is adopted by many others studies. However, this type of psychological questionnaire is criticized for its tendency to acquiescence, since subjects were answering the questions by supporting rewarding answers which they think are expected by the experimenter. It is the positive which socially desired ad was favored [27]. Thus, the subject minimizes perhaps unconsciously, the difficulties required to build a self-image.

In addition, it is important to specify that MI ability depends on other factors such as noise, anxiety but also on the level of training. Sports studies established that it is easier to mentalize a gesture for an experienced athlete compared to a beginner [28, 29]. Recently, Yasemidou et al. have tested an interactive 3D models of task-relevant anatomy to be used in conjunction with mental imagery to facilitate generation of mental images. According to this pilot study, the use of 3D interactive model might enhance the imagery quality and subsequently optimize surgical performance [30].

There are few studies that have evaluated the value of MI in surgical learning. A review of the literature published in 2015 identified 9 randomized control studies over reported randomized control studies [31–41]. Five of them objectified a beneficial effect of MI on the learning of surgical technical skills while four studies were negative. A meta-analysis based on this population showed the difficulty to demonstrate a superiority of MI [22]. However, the results of this meta-analysis should be interpreted with high caution since the studied populations as well as the assessment tools used were extremely heterogeneous [22]. For example, neither the length nor the time between the MI session and the assessment were standardized in most discussed studies. A systematic review published in 2017 collecting 14 studies showed that MT is beneficial in surgical education in the majority of studies with a ratio of 11 out 14 [42]. However, authors have remarked that overall studies were low quality, lacking a sufficient methodology and suffered from small sample sizes issue. As a result, they have concluded that further studies are needed to identify the best role for MI as a supplementary instructional method in the surgical curriculum. Another, recent systematic review published in 2020 including 7 sound peer-reviewed study published between 2010 and 2020 suggested that mental practice improved simulation and performance on operating room, however authors highlighted that there is insufficient evidence to intermediate or long term efficacy of Mental practice [43]. Table 3 illustrates the results of the main reported studies in the literature.

**Table 3:** Mental imagery research in surgery

Study	Population	Surgical skills	Findings
Jungmann et al. 2011 [37]	40 medical students	Laparoscopic knots	NS
Donnon et al. 2005 [40]	42 medical students	Laparoscopic suture	NS
Mulla et al. 2012 [38]	41 medical students	cutting a circle ON pelvi-trainer	NS
Immenroth et al. 2007 [33]	98 novices surgeons	LC	S
Komesu et al. 2009 [41]	64 novices surgeons	Cystoscopy	S
Arora et al. 2011 [35]	18 novice et expert surgeons	VR LC	S
Vignes et al 2013 [44]	40 medical students	Surgical hand-washing	S
Sanders et al. 2008 [34]	65 medical students	Suture on pigs’ feet	S
Arora et al. 2010 [35]	20 novice’s surgeons	LC	S
Eldred-evans et al. 2013 [39]	64 medical students	cutting a circle drawn on stretchable material	S
Arora et al. 2011(b) [36]	18 novices surgeons	Surgical stress	IM decreases surgical stress
Conlin et al. 2016 [45]	13 ORL residents	mastodectomy	NS
Saab et al. 2017 [46]	20 surgeons	Abdominal hysterectomy	S
Geoffrion et al. (2012) [47]	Junior gynecology resident	Vaginal hysterectomy	NS
Louridas et al. (2015) [48]	Senior surgical trainees	Procine laparoscopic jejunostomy	S

The impact of MI on surgical stress is another facet that has been recently explored by several authors. In fact, we know that excessive stress is adversely affecting the cognitive processes including memory, attention, and concentration, but also performance of complex motor skills [49–51]. In surgical field, it was evidenced that surgeons are exposed to excessive levels of stress in the operating room [51–53]. Consequently, this impacts the motor skills, surgical decision-making, surgical performance and patient safety. A prospective, randomized controlled study of 20 novice surgeons has evaluated how MI affects the surgical stress management [33]. The task performed was based on simulator laparoscopic cholecystectomy (LC). Stress was assessed subjectively using a validated version of State-Trait Anxiety- Inventory (STAI) questionnaire and objectively with a continuous heart rate (HR) monitoring and salivary cortisol. Mental imagery was assessed using a validated mental imagery questionnaire. This study suggested that a short prior session of mental imagery reduced the psychological, cardiovascular and neuroendocrine response to stress during simulator surgery task.

Vignes et al. studied mental imagery in novices using a similar methodology to ours [44]. It consisted of evaluating the interest of MI in learning of surgical washing in 64 medical students randomized into two groups of “MI” and “control”. The evaluated criteria include surgical growing, surgical hand-washing technique and the application of hydro-alcoholic solution. The results of this study are supporting the superiority of MI. However, it should be noticed that this study was comparing the overall score, while the results of different items were not evaluated. A reported work by Sanders et al. was evaluating MI in second year medical students that completely new in surgery. They compared mental imagery review and classic review using paper documents. They demonstrated a benefit MI [21]. Among the negative studies in novices, we cite Mulla et al. which compared MI with pelvitrainer training and simulator training in 41 medical students [25]. The used exercise consisted of cutting a circle drawn on stretchable material (a glove). The results obtained in the assessment using IM group are the worst among the 3 groups. The authors then conclude that MI cannot replace traditional training which remains compulsory for novices.

In our study, we have opted for HIA as a gestural procedure to evaluate. It is a multi-step gesture requiring both finesse and concentration and very high cognitive solicitation. The tested gestures in the literature vary from a basic gesture including simple suture and laparoscopic knots, to a more complete intervention including laparoscopic cholecystectomy. Indeed, whatever was the chosen gestural model, the most important is the protocolization of the surgical procedure and the definition of the evaluation aspects. In our study, we have divided the studied surgical gesture into 7 sequences. Thus, a well-defined explicit protocol allows a better assimilation of the surgical gesture and consequently an optimization of its mentalization. This is the most interesting aspects for novice phase of learning.

Although the overall score obtained was significantly higher in the MI group of our results, it should be noticed that the differences were only significant for 6 items out of 14. While we did not observe any beneficial effect for the remaining items. In fact, we believe that items which are not reflecting the superiority of the MI, are often complex or requiring highly automated actions that involve a great deal of experience from the surgeon. To explain the lack of benefit of MI on these more complex gestures, we refer to one of the classic learning theories of motors skills, learning describing the profile of our situation [54]. According to this theory, gesture learning can be broken down into 3 phases. During the first stage known as the cognitive or imitation phase, the learner receives verbal written or schematized information guiding the performance of the technique, this is the stage where errors occur. The second stage is called associative, during which the learner corrects his errors until assimilating the taught technique. The third and final stage is the autonomy where the learner is expressing to master perfectly the taught technique with higher mechanization and the performed steeps are almost achieved “unconsciously”. It seems that the actual occurrence of errors and their correction is a necessary and valuable step in the learning process of complex gestures for a novice learner. Hence there is crucial role of supervised hands-on. Briefly, MI could speed-up the acquisition of certain gestural skills without substituting companionship for novice learners in the operating room. It is also important to notice that the best way to implement MI in surgical skill course is a matter of considerable debate in the literature. In a pilot study, evaluating the efficacy of MI in 20 trainees on Laparoscopic Suturing and Knot tying, the authors did not found any benefit and suggest that it should not be implemented for novice surgeons. According to authors, cognitive training should be more adapted for intermediate to advanced surgeons to strengthen already acquired and memorized techniques (in particular, those related to spatial abilities) [55]. Otherwise, a systematic review of the literature performing a real benchmarking including wide range of studies in several MI fields suggested that a successful MI training programs could be delivered in parallel to existing surgical training, in a flexible format allowing surgeons to undertake several MI sessions in a self-directed manner [56].

Finally, despite many limitations, our results are opening an interesting educational research path. Further extension of this study is planned to implement a more robust method while including larger size sample, more homogeneous population and a longer duration of MI session. It will also be necessary to use tools to assess the MI ability of each participant in order to ensure its efficacy.

## Conclusion

Our study demonstrated that the use of mental imagery immediately after a learning session improves a selected gestural performance in novice surgeons. In view of these results, mental imagery appears to be a promising, self-replicating and inexpensive educational approach deserving more significant role in modern surgical education setup. Besides, studies with advanced design and methodological setup would better elucidate the best further protocol elaboration to integrate MI in surgery education program in respect with rules of good surgery practice.

## Data Availability

The datasets used and/or analyzed during the current study available from the corresponding author on reasonable request.
